# Dislocation of the Thigh, and Reduction by the Patient

**Published:** 1851-06

**Authors:** B. L. Delano


ART. II.—*Dislocation of the Thigh, and reduction by the Patient.*—Communicated by B. L. DELANO, M. D.

DR. FLINT,—DEAR SIR:

I herewith transmit a transcript of a letter from Marcus Swain, M. D., a reputable practitioner of Essex, Vt., detailing the particulars of an unfortunate occurrence which obtained with his own person recently; the result of which seems to me so remarkable, as to deserve a circulation in your useful journal.

Though perhaps it may be feasible to reduce a luxated hip-joint in the manner described by Dr. Swain, the practice certainly is modern, and thus far has been adopted, but to a limited extent; but that the patient should be the operator, without even the aid of an assistant, is a feature that renders the case one of unusual interest; and although I have not the permission of the Doctor to publish his case of surgery, or blazon abroad his superlative tact as a *bone setter*, yet the extraordinary character of this case I claim as justification for so doing, and I am disposed to take upon myself the responsibility; and, if my old "*chum*" has any complaints to render, he knows full well upon whom rests his claim. *

LOCKPORT, April 24, 1851.

"Friend Delano: * * * * "I had the misfortune about the twentieth of February last, to slip down upon the ice, and dislocate my left hip joint, which completely disabled me for several weeks, and have not yet entirely recovered from the effects of the injury. I have, however, so far recovered as to be able to walk with the assistance of a cane, and I have now resumed the duties of my profession. There is some interest connected with the case; and I will briefly give you a history of it. It being very icy, and raining hard at the time, I took my umbrella in one hand, and my *pill bags* upon the other arm, and started to visit a patient in the village, it being about eight o'clock in the evening; while walking upon the ice rather fast, my feet tripped, and I fell with great force upon my left hip, bruising mainly the region of the trocanter major, and upper part of the femur. I felt the slipping of the head of the femur as it passed out of the acetabulum; and I assure you, the pain attending it was of an acute and lancinating kind; perhaps never before felt by me. I immediately attempted to rise, but could not: on getting upon my hands and right knee, I attempted to bring my left limb forward, but could not: it remained in an extended position, and I had little or no power over it. I then seized the left limb with my left hand, and flexed the thigh forward upon the pelvis; and in doing this, with considerable force, I reduced the head of the bone to its proper place.

*This I consider to be the most fortunate part of the accident.* I claim no peculiar skill in reducing the bone so readily; I admit it to be rather accidental; yet, I have been led to reflect upon accidents of this nature, and am inclined to think that in many cases, and perhaps in all, they may be reduced by a motion of this kind; that is, by flexing the leg upon the thigh, and the thigh upon the pelvis,— with care, yet with a determined firmness. Very recently I have been informed that Prof. Moore, of Woodstock Medical College, recommended this plan to his class last spring. If the practice should be found to work well, and enable us to dispense with so much extension, and counter-extension, the use of pullies, &c., I think an important point would be gained to the patient certainly, if not to the surgeon. What think you of the suggestion? * * * *

Please write at your earliest convenience, and believe me,

Truly yours, M. SWAIN.

Essex, Vt., April 18, 1851."

